# Quality Assessment of Hypertension Treatment–Related Information on WeChat: Cross-sectional Study

**DOI:** 10.2196/38567

**Published:** 2022-10-26

**Authors:** Yuting Yang, Mengchi Hou, Xue Gong, Rui Guo, Xing Lin Feng, Rui Tian

**Affiliations:** 1 School of Public Health Capital Medical University Beijing China; 2 China Aerospace Science & Industry Corporation 731 Hospital Beijing China; 3 Department of Health Policy and Management, School of Public Health Peking University Beijing China; 4 Capital Medical University Library Capital Medical University Beijing China

**Keywords:** quality assessment, hypertension, WeChat, DISCERN instrument

## Abstract

**Background:**

The WeChat platform has become a primary source for medical information in China. However, no study has been conducted to explore the quality of information on WeChat for the treatment of hypertension, the leading chronic condition.

**Objective:**

This study aimed to explore the quality of information in articles on WeChat that are related to hypertension treatment from the aspects of credibility, concreteness, accuracy, and completeness.

**Methods:**

We searched for all information related to hypertension treatment on WeChat based on several inclusion and exclusion criteria. We used 2 tools to evaluate information quality, and 2 independent reviewers performed the assessment with the 2 tools separately. First, we adopted the DISCERN instrument to assess the credibility and concreteness of the treatment information, with the outcomes classified into five grades: *excellent*, *good*, *fair*, *poor*, and *very poor*. Second, we applied the Chinese Guidelines for Prevention and Treatment of Hypertension (2018 edition) to evaluate the accuracy and completeness of the article information with regard to specific medical content. Third, we combined the results from the 2 assessments to arrive at the overall quality of the articles and explored the differences between, and associations of, the 2 independent assessments.

**Results:**

Of the 223 articles that were retrieved, 130 (58.3%) full texts were included. Of these 130 articles, 81 (62.3%) described therapeutic measures for hypertension. The assessment based on the DISCERN instrument reported a mean score of 31.22 (SD 8.46). There were no articles rated *excellent* (mean score >63); most (111/130, 85.4%) of the articles did not refer to the consequences—in particular, quality of life—of no treatment. For specific medical content, adherence to the Chinese Guidelines for Prevention and Treatment of Hypertension was generally low in terms of accuracy and completeness, and there was much erroneous information. The overall mean quality score was 10.18 (SD 2.22) for the 130 articles, and the scores differed significantly across the 3 types (*P*=.03) and 5 sources (*P=*.02). Articles with references achieved higher scores for quality than those reporting none (*P*<.001). The results from the DISCERN assessment and the medical content scores were highly correlated (ρ=0.58; *P*<.001).

**Conclusions:**

The quality of hypertension treatment–related information on the WeChat platform is low. Future work is warranted to regulate information sources and strengthen references. For the treatment of hypertension, crucial information on the consequences of no treatment is urgently needed.

## Introduction

### Background

Hypertension is a public health challenge because of its high risk for cardiovascular disease, which is the top cause of morbidity and mortality worldwide [1,2]. In 2019, an estimated 1.28 billion adults aged 30 to 79 years worldwide—approximately 32% to 34% of the global population—were diagnosed with hypertension [3]. In China, nearly half of the adults aged 35 to 75 years were diagnosed with hypertension as of 2017, with medication adherence and control rates <50% and <20%, respectively [4]. Hypertension is a chronic condition that needs lifelong treatment, and the treatment includes two aspects: health management (condition monitoring, lifestyle intervention, control of complications, etc) and taking medications to control rising blood pressure [5,6]. It has been revealed that awareness is the first step for devising appropriate management [7], with the detection rate a key factor that affects treatment and control [8,9]. For patients who are aware of their condition, treatment-related information, including general illness information and treatment choices, is a major concern. Patients also want to play a more active role in decision-making to ease anxiety [10]. Nevertheless, they usually encounter difficulties in finding relevant and easy-to-understand information.

The internet is the first source of medical information for the public as well as patients because of its speed and cost-effectiveness [11]. Toward the end of 2021, China had 1.03 billion internet users, the largest population of *netizens* in the world [12]. WeChat is the primary social media platform for Chinese netizens, equivalent to Facebook for other international community members and providing similar service models. Social media platforms have a convenient search function. On the basis of related keywords, one can retrieve articles, videos, and almost anything one wants [13]. WeChat was launched in 2011, and by June 2022, the monthly WeChat active users had reached 1.3 billion. With >700,000 articles posted daily [14], WeChat has become the most important information source for the Chinese public. Zhang et al [15] found that 98.35% of the participants reported that they had seen health information via WeChat, and WeChat was one of the most popular choices (63.26%) for obtaining health information in China. Despite the benefits, the health information obtained via WeChat has some limitations, with concern about information quality being the most mentioned [16]. On WeChat, the information sources are numerous and unclear, which has resulted in problems of questionable credibility and inaccuracy [17]. Meanwhile, the health literacy of the general population in China is low [10], because of which low-quality health information can lead to harmful behavior. Therefore, it is critical to evaluate the quality of hypertension-related information on WeChat. We found that only 1 study had been conducted to assess the quality of hypertension-related information provided on traditional websites [18]. However, no studies are available assessing the quality of hypertension-related information on WeChat.

### Objectives

DISCERN is the most widely used instrument for assessing health-related information and videos, and it is particularly relevant to health-related topics and web-based resources for patient education [19]. Literature is emerging that combines the results from the DISCERN tool and other ratings of web-based references on specific professional content based on clinical guidelines [18,20,21]. This study aimed to assess the quality of information in hypertension treatment–related articles oriented to the general population on WeChat. We adopted the DISCERN instrument to assess the credibility and concreteness of the treatment information and then applied the Chinese Guidelines for Prevention and Treatment of Hypertension (2018 edition; hereinafter referred to as the Hypertension Guidelines) [22] to evaluate the accuracy and completeness of the specific medical content of the treatment information. We combined the results from the 2 sources to report on the overall quality of the articles to comprehensively evaluate the quality of information on WeChat. We believe that this is the first report on the quality of information in hypertension treatment–related articles on WeChat.

## Methods

### Search Strategy and Data Extraction

In this study, we entered the terms “高血压治疗 (hypertension treatment),” “高血压疗法 (hypertension therapy),” “高血压防治 (hypertension prevention and treatment),” “高血压健康干预 (hypertension health intervention),” and “高血压健康管理 (hypertension health management)” into WeChat for retrieval of relevant articles. To ensure that the articles included in the study matched the research aims, we used certain inclusion and exclusion criteria. The inclusion criteria were as follows: (1) articles focusing on information related to hypertension treatment and health management and (2) articles covering hypertension treatment and therapy. The exclusion criteria were as follows: (1) duplicated articles, (2) articles providing full texts from the Hypertension Guidelines, and (3) articles presented only in picture or video format.

**Figure 1:**
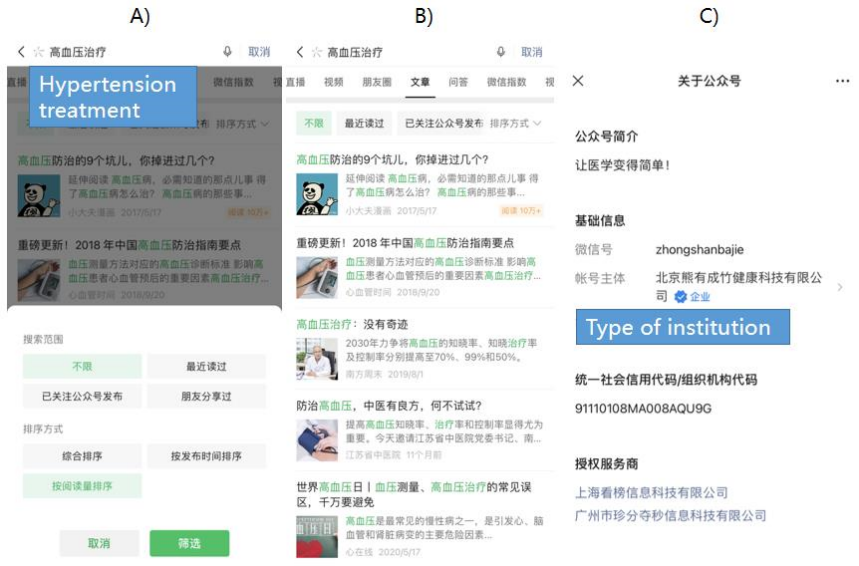
Examples of the search for articles in WeChat public accounts. (A) Retrieval strategy for hypertension treatment related to the keyword “高血压治疗 (hypertension treatment)” in the WeChat app. (B) Retrieval results. (C) Information provided in the WeChat public accounts. Retrieval date: May 30, 2021.

**Figure 2:**
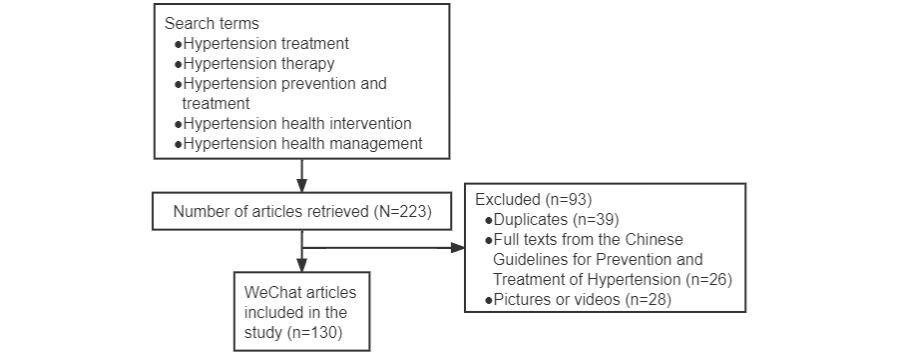
Search and screening flow for hypertension treatment–related articles.

We included relevant data in this study up to the date of information collection, namely May 30, 2021. In Figure 1, we provide some examples of the retrieval strategy and results. After the screening, we included 130 articles for further data extraction and analysis. Figure 2 illustrates the search and screening flow for the articles. We extracted the essential information for each article and its source, including the article title, publication date, numbers of views and *likes*, type of treatment mentioned, uploader (governmental organization vs individual, etc), and references. The extracted data were recorded in Excel (Microsoft Corp).

### Ethical Considerations

Institutional review board approval was not required for this study since all information was freely available online. The “articles” were defined as being any piece of open access published writing, excluding personal blogs, editorials, and commentaries.

### Evaluated Dimensions and Methods

#### Overview

**Figure 3:**
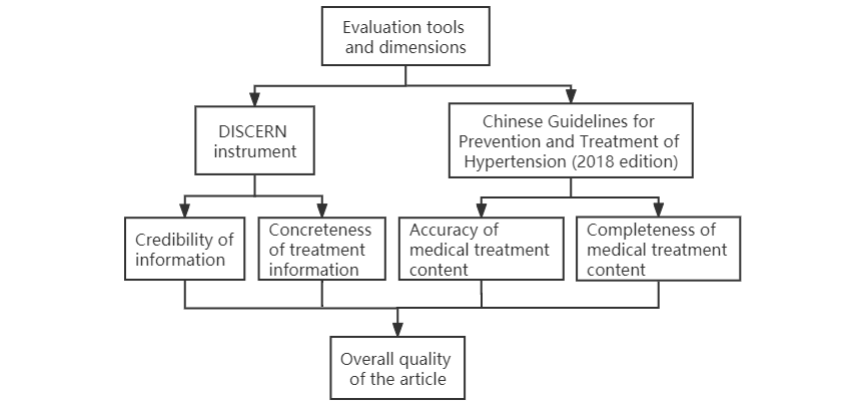
Evaluation tools and dimensions.

We measured two aspects of hypertension treatment–related articles on WeChat: the quality of information and the content, to evaluate which we used 2 metrics. First, we adopted the DISCERN instrument, which assesses the credibility and concreteness of written consumer health information with regard to treatment choices. Second, we applied the Hypertension Guidelines as a supplement to evaluate the accuracy and completeness of the specific medical content in the article, which provided a more granular assessment of the quality of information as it pertains to hypertension treatment. We provide details of the tools in the sections that follow. Third, we combined the results from the 2 assessments to arrive at the overall quality of the articles and explored the differences between, and associations of, the 2 independent assessments (Figure 3). Two researchers assessed each article and scored both instruments. The intraclass correlation coefficient (ICC) was used to measure consistency [23]. The ICC value is between 0 and 1. It is generally acknowledged that ICC>0.80 indicates strong consistency, ICC from 0.80 to 0.41 indicates medium consistency, ICC<0.40 indicates poor consistency, and ICC<0 is considered no consistency [24,25].

#### DISCERN: Assessment of Credibility and Concreteness of Treatment Information

To rate the quality of the information, we adopted the DISCERN instrument. The DISCERN handbook indicates that both professionals and the general population can use the instrument, and a study has confirmed that professionals judge health information similarly to laypersons using DISCERN [26]. The handbook is available on the DISCERN website.

The instrument consists of 16 questions divided into 3 parts (the overall score ranges from 16 to 80) [26]. Part 1 (questions 1 to 8) assesses the credibility of the information, part 2 (questions 9 to 15) focuses on the concreteness of treatment information, and part 3 (question 16) is an overall quality rating [27]. In this study, an article could score up to 80 points on all 16 questions, with up to 40 points for the questions addressing the credibility of information (questions 1-8), up to 35 points for the questions addressing treatment choices (questions 9-15), and up to 5 points for question 16. High scores indicate high quality. For describing and distinguishing the DISCERN scores significantly, we adopted the approach used in a previous study and categorized scores of 63 to 80 as *excellent*, 51 to 62 as *good*, 39 to 50 as *fair*, 27 to 38 as *poor*, and 16 to 26 as *very poor* [18,28]. YY and MH performed the scoring and used the ICC to measure consistency.

#### Hypertension Guidelines: Assessment of Accuracy and Completeness of Medical Treatment Content

The DISCERN tool can be used for any health-related content area and, thus, is not specific to hypertension [29]. Therefore, we used the Hypertension Guidelines as a supplement to evaluate the accuracy and completeness of the specific medical content in the included articles. We referred to the DISCERN scoring criteria and developed the content evaluation criteria to maintain consistency and comparability with the DISCERN tool [30]. With regard to accuracy, we chose the following categories: completely accurate (5 points), partially accurate (3-4 points), not very accurate but containing no errors in the information (2 points), and wildly inaccurate and containing misinformation (1 point) [25]. For completeness, the Hypertension Guidelines mentions 6 aspects of hypertension treatment [22]: (1) hypertension treatment goals, (2) lifestyle intervention, (3) medical treatment, (4) instrument intervention, (5) management of related risk factors, and (6) treatment of hypertension in special populations. On the basis of the coverage of these 6 key points, we developed the following categories: all 6 key points mentioned (5 points), 4 to 5 key points (4 points), 3 key points (3 points), 1 to 2 key points (2 points), and no mention of any of the key points (1 point). YY and MH performed the scoring and used the ICC to measure consistency.

### Overall Article Quality

In general, we combined the DISCERN tool and the Hypertension Guidelines to measure the overall quality of the article. First, we calculated the mean scores of part 1 and part 2 of the DISCERN tool with regard to the credibility and concreteness of information about treatment choices, respectively. Second, we used the 2-part mean scores of accuracy and completeness for the medical treatment content evaluation, reflecting the quality specifically for hypertension. Then, we added the 4-part scores to arrive at the overall quality score for exploration of the quality differences (Figure 3).

### Exploring the Quality Differences

Considering the different value propositions, for the comparison, we identified 3 categories of articles, 5 types of sources, and 2 kinds of articles according to whether there were references. First, we combined the treatment aspects of the Hypertension Guidelines and divided the articles into 3 categories: (1) therapeutic measures, (2) lifestyle intervention, and (3) scientific or frontier knowledge (introduction of new drugs, etc). Second, we classified the articles’ uploaders into five main categories: (1) governmental organizations, (2) commercial organizations, (3) medical institutions, (4) news or media organizations, and (5) individuals. Third, we divided the articles into 2 kinds according to whether there were references. The purpose was to explore the differences between them in terms of article quality. Detailed information of each uploader was shown in its public account, including the name, time of upload, and institution type (Figure 1).

### Statistical Analysis

We used Excel 2019 (Microsoft Corp) for data collection and SPSS software (version 26.0; IBM Corp) for analysis. Data were presented as frequencies and percentages or means and SDs as appropriate. Regarding the evaluation scores, we used the ICC to ascertain the interrater agreement with regard to the exploration of quality differences. Kruskal-Wallis tests were used to determine statistically significant differences between 2 groups or among >2 groups of independent variables. The correlations among the DISCERN scores, content scores, number of views, and number of *likes* were evaluated using Spearman correlation analysis. *P*<.05 was considered statistically significant.

## Results

### Characteristics of the Articles

**Figure 4:**
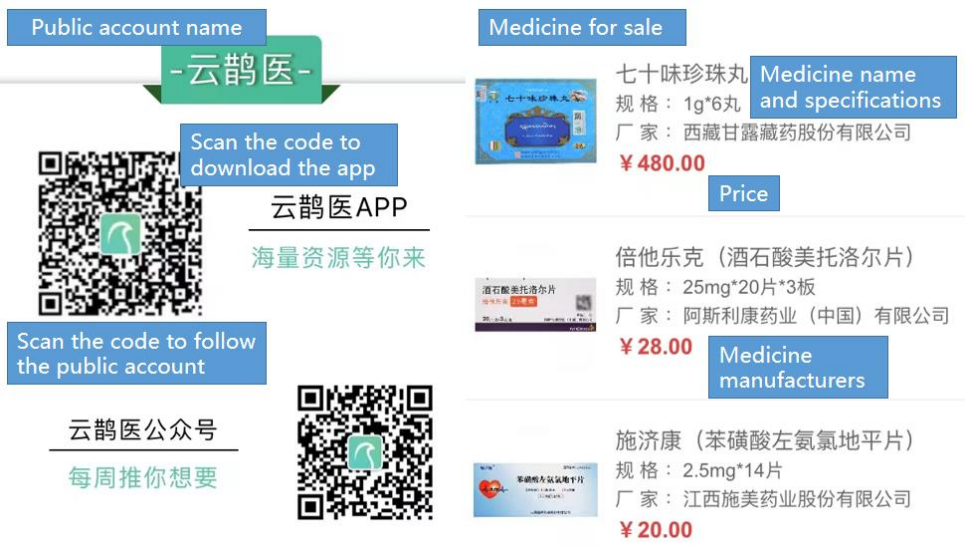
Examples of different marketing strategies appended to the end of articles.

In this study, the search retrieved 223 articles, of which we included 130 (58.3%) for analysis according to the inclusion and exclusion criteria (Figure 2). In terms of the treatment information types, 62.3% (81/130) of the articles related to therapeutic measures, 26.9% (35/130) referred to lifestyle intervention, and 10.8% (14/130) involved scientific or frontier knowledge (Table 1). With regard to the uploading source, the majority of the articles had been posted by commercial organizations (78/130, 60%), followed by individuals (29/130, 22.3%), medical institutions (10/130, 7.7%), news or media organizations (9/130, 6.9%), and governmental organizations (4/130, 3.1%; Table 1). Only 13.1% (17/130) of the articles provided references. In addition, 89.2% (116/130) of the articles adopted various marketing strategies for promotion of the content. In Figure 4, we provide examples of these different marketing strategies.

With regard to the number of views, the median was 2929 (range 7 to >100,000); the minor articles (1/130, 0.8%) had been read only 7 times, and only 0.8% (1/130) of the articles had been read >100,000 times. In terms of the *likes* received, the median was 9.5 (range 0-951); >1 article had not received a single *like*, whereas the highest number of *likes* for an article was 951 (Table 1).

**Table 1:** Characteristics of articles related to hypertension treatment on WeChat (N=130).

Variable					Values		
**Category, n (%)**							
	**Information category**						
		Therapeutic measures				81 (62.3)	
		Lifestyle intervention				35 (26.9)	
		Scientific or frontier knowledge				14 (10.8)	
	**Uploading source**						
				Commercial organizations		78 (60)	
				Individuals		29 (22.3)	
				Medical institutions		10 (7.7)	
				News or media organizations		9 (6.9)	
				Governmental organizations		4 (3.1)	
	**Reference source**						
			Yes			17 (13.1)	
			No			113 (86.9)	
**Metrics, median (range)**							
	Number of views						2929 (7-100,000)
	Number of *likes*						9.5 (0-951)

### Evaluated Results

#### DISCERN: Information Credibility and Concreteness of Treatment Information

The complete table with the average scores of the 130 articles derived by using the 16 questions of the DISCERN instrument is available in Multimedia Appendix 1. Overall, the quality was poor based on the credibility and concreteness of treatment information. According to the DISCERN scale, no article was *excellent* in terms of the information provided; 3.1% (4/130), 18.5% (24/130), and 44.6% (58/130) of the articles were *good*, *fair*, and *poor*, respectively. Besides, 33.8% (44/130) of the articles obtained an abysmal score. An article could score up to 80 points on 16 questions, but the mean score of these 130 WeChat articles was 31.22 (SD 8.46; median 30.00; range 16-58). The ICC was between 0.69 and 0.97, indicating an acceptable consistency.

When part 1 (questions 1-8) and part 2 (questions 9-15) scores are compared, the former comes off slightly better than the latter. In part 1, the article’s information credibility was evaluated and received a mean score of 16.58 (SD 4.86). “Are the aims clear?” (question 1) received the highest mean score: 2.87 (SD 0.76). “Does it refer to areas of uncertainty?” (question 8) performed the worst with a mean score of 1.38 (SD 0.79). Part 2 assessed the concreteness of treatment information; the mean score was 12.25 (SD 3.85), indicating that the information provided on treatment choices was generally poor, and this was particularly notable in “Does it describe how the treatment choices affect the overall quality of life?” (question 13), which received a mean score of 1.18 (SD 0.46). Multimedia Appendix 1 shows the detailed scores.

#### Hypertension Guidelines: Accuracy and Completeness of Medical Treatment Content

With regard to the accuracy and completeness of the hypertension treatment–related medical content, the former performed better than the latter. The ICCs were 0.77 and 0.86, respectively, indicating an acceptable consistency. With regard to article accuracy, the average score was 3.43 (SD 0.79); we found that some (3/130, 2.3%) of the articles contained typographical errors, erroneous information, or areas of content that needed improvement. For completeness, the average score was 2.94 (SD 0.81); most (98/130, 75.4%) of the articles lacked key points on hypertension treatment content according to the Hypertension Guidelines, resulting in the incompleteness of content. In Table 2, we provide some typical examples of content deficiencies or scientific content inaccuracies and suggestions for improvement. Table 3 shows the detailed scores.

**Table 2:** Typical examples of assessment of the content of some articles on hypertension.

Article title	Areas of deficiencies	Suggestions for improvement
Hypertension prevention and treatment: traditional Chinese therapy has a good remedy, why not try it?	The title does not match the content. The title emphasized traditional Chinese therapy, whereas the content mainly focused on Western medicine. There was no significant description of how each therapy works, and there was a lack of concrete critical information, reducing the effect and meaningfulness of the article.	As the title of the article emphasizes the benefit of using traditional Chinese therapy to treat hypertension, the content should match the title, and the article should describe traditional Chinese therapy in detail. The article should provide detailed descriptions of drugs and therapies, clarifying the effects of using traditional Chinese therapy and indications for its use; for example, how it works, the benefit, the risk of each treatment, and what would happen if no treatment is used.
How is the hypertension treatment plan developed?	Hypertension treatment is primarily based on the Chinese Guidelines for Prevention and Treatment of Hypertension, but concreteness was lacking. For hypertension medical treatment, the article only provided a brief statement. The article did not indicate the source of information.	Copying and presenting the Chinese Guidelines for Prevention and Treatment of Hypertension is a good idea, but it is better to extract concrete information related to the main idea of the article, keeping in mind the completeness of key content. Indicating the source of information is good practice and so is providing a link to the reference in the article, as is done in research papers.
Treatment of hypertension (posted by an individual, Xuejie Han)	The article uses colloquial language, it is verbose, and it even contains wrongly written characters. With regard to the source, the article indicates that “This text is from the network,” but the lack of specifics only raises questions about the scientific and safety issues of the content.	The writer should avoid using colloquial language, keep sentences simple, and be serious about avoiding incorrect and inaccurate words. It is better to reference authoritative books, papers, or resources.

**Table 3:** The mean scores of the articles as evaluated using the DISCERN instrument and the Chinese Guidelines for Prevention and Treatment of Hypertension.

Content evaluated	Scores, mean (SD)
Part 1 (credibility of information)	2.07 (0.61)
Part 2 (concreteness of treatment information)	1.75 (0.55)
Accuracy of treatment information^a^	3.43 (0.79)
Completeness of treatment information^b^	2.94 (0.81)
Overall quality	10.18 (2.22)

^a^Intraclass correlation coefficient=0.77.

^b^Intraclass correlation coefficient=0.86.

#### Overall Article Quality

First, in terms of the mean DISCERN scores, part 1 (credibility of information) and part 2 (concreteness of treatment information) scored 2.07 (SD 0.61) and 1.75 (SD 0.55), respectively. Second, in terms of the mean Hypertension Guidelines scores, the accuracy and completeness of the medical treatment content scored 3.43 (SD 0.79) and 2.94 (SD 0.81), respectively. Third, the mean score of the overall quality of the articles was 10.18 (SD 2.22; Table 3).

### Comparison of Treatment Information Types, Uploading Sources, and Availability of References

We identified 3 categories of articles and 5 types of sources and divided the articles into 2 kinds according to whether they provided references. We chose the overall quality, DISCERN, and medical content scores for comparison. First, there were significant differences among the 3 types (*P*=.03), primarily because of the differences in medical treatment content quality (*P*=.02). Second, statistically significant differences could be observed in the overall quality among the 5 sources (*P*=.02), mainly because of the differences in DISCERN-evaluated quality (*P*=.02). By contrast, there were no statistically significant differences in the medical content quality scores (*P*=.10). Governmental institutions scored the highest (mean 11.01, SD 1.36), and individuals scored the lowest (mean 9.08, SD 2.13). Table 4 shows the results. Third, we compared the articles’ quality in terms of whether they provided references, and the results showed statistically significant differences (*P*<.001) between articles that provided references and those that did not. The mean score of articles that provided references was significantly higher than those that did not, 12.90 (SD 1.83) and 9.78 (SD 1.81), respectively.

**Table 4:** Comparison of the DISCERN scores of information categories, uploading sources, and reference sources.

Item		Score, mean (SD)^a^	*P* value^b^		*P* value^c^		*P* value^d^	
**Information category**				.03		.42		.02
	Therapeutic measures	10.51 (2.44)						
	Scientific or frontier knowledge	9.91 (2.14)						
	Lifestyle intervention	9.54 (1.58)						
**Uploading source**				.02		.02		.10
	Governmental institutions	11.01 (1.36)						
	News or media organizations	10.87 (1.34)						
	Commercial organizations	10.47 (2.35)						
	Medical institutions	10.19 (1.50)						
	Individuals	9.08 (2.13)						
**Reference source**				<.001		<.001		<.001
	Yes	12.90 (1.83)						
	No	9.78 (1.81)						

^a^The Kruskal-Wallis test was used as a conservative test when determining significance for continuous variables, given the nonnormality of some data. The mean (SD) values refer to the overall quality score.

^b^*P* value is applicable to the overall quality score.

^c^*P* value is applicable to the mean score of DISCERN part 1 and part 2 assessments.

^d^*P* value is applicable to the mean score of accuracy and completeness of medical treatment content.

### Quality Assessment and Correlation With Numbers of Views and Likes

Significant correlations were observed between the DISCERN score and the medical content score (*P*<.001). This demonstrated that if the credibility and concreteness of treatment information were excellent, the medical accuracy, completeness, and overall quality were likely to be better; in other words, the DISCERN tool and the Hypertension Guidelines can validate each other. Meanwhile, there was a significant correlation among credibility and accuracy, the concreteness of treatment information, and completeness, which revealed that the evaluations performed using the corresponding parts of the 2 tools were consistent. By contrast, there was no significant correlation among the DISCERN score, the number of views (*P*=.63), and the number of *likes* (*P*=.23); the content score results were similar (*P*=.10 and *P*=.11), which means a good-quality article does not necessarily receive a high number of views (Table 5).

**Table 5:** *P* values of correlation of DISCERN score, content score, and numbers of views and likes.^a^

	DISCERN score	Content score	Number of views	Number of *likes*
DISCERN score	<.001^b^	—^c^	—	—
Content score	<.001^d^	<.001^b^	—	—
Number of views	.63	.10	<.001^b^	—
Number of *likes*	.23	.11	<.001^e^	<.001^b^

^a^Credibility and accuracy: *P*<.001; concreteness of treatment information and completeness: *P*<.001.

^b^Spearman correlation coefficient=1.00

^c^Not applicable.

^d^Spearman correlation coefficient=0.58.

^e^Spearman correlation coefficient=0.63.

## Discussion

### Principal Findings

This study has provided the first report on the quality of information in hypertension treatment–related articles on WeChat. The evaluation outcomes from the two sources, that is, the DISCERN and the Hypertension Guidelines, show high correlations and suggest valid results. The overall quality of hypertension treatment–related information on WeChat was poor in terms of credibility, concreteness, accuracy, and completeness. Quality scores differed significantly among the 3 types of articles and 5 information sources, revealing the significance of different value propositions. Articles reporting references were of better quality than those that did not provide references. Our findings and methods have important implications in an era when people increasingly use social media to obtain health-related information.

### Comparison With Prior Work

Prior studies have investigated the quality of web-based information with regard to different diseases and platforms; for example, the study by Azer et al [31] evaluated the quality of information on the internet about inflammatory bowel disease (mean 42.2, SD 10.7), and the study by Kaicker et al [27] evaluated the quality of information on chronic pain (mean 55.9, SD 13.6). We only discovered 1 study evaluating hypertension-related information quality on websites, with a DISCERN score of 45.94, and only 1 of these websites was excellent [19]. The score was higher than ours, probably because WeChat’s articles were more subjective and had been uploaded by random sources [31]. With regard to social media platforms, researchers have investigated the quality of YouTube videos about eczema treatment (mean 30.6) and meningioma treatment (mean 36.4, SD 14.0) [32,33], as well as the quality of treatment of rare diseases on WeChat (mean 30.27, SD 7.20). These scores are similar to that obtained in this study (mean 31.22, SD 8.46). Overall, websites performed better than social media platforms. This might be because user barriers as well as barriers to publishing on social media platforms are low because these platforms encourage everyone to participate and share content [34]. However, we must consider the particularities of medical health information, which differs from other types of information [35]. Therefore, it is essential to encourage medical professionals, scientific researchers, and those who have received professional certification to provide health-related content and articles. These people should do more to popularize science and meet the general population’s needs.

### Articles of Good or Excellent Quality Are Rare

Generally, the quality of hypertension treatment–related information on WeChat was found to be poor in terms of credibility, concreteness, accuracy, and completeness, which is not helpful for the general population. In the DISCERN evaluation, no article was found to be *excellent*, and only 3.1% (4/130) of the articles were rated *good*. This finding is consistent with prior studies on health information on websites and YouTube; for example, the study by San Giorgi et al [36] found that only 2% of the websites evaluated provided *good* content, and the study by Śledzińska et al [33] found that only 4.9% of the YouTube videos assessed were rated *good*. Our study revealed that the credibility of information generally scored higher than the concreteness of treatment information. In other words, compared with the article’s credibility, the concreteness of treatment information is harder to achieve. With regard to credibility, “Are the aims clear?” (question 1) received the highest mean score, which was probably related to the emphasis on patient-centered health information services and the need for clear goals in the process of information dissemination [37,38]. This revealed that the match of title and content of hypertension treatment–related information is relatively acceptable. “Does it refer to areas of uncertainty?” (question 8) performed the worst; although most (100/130, 76.9%) of the articles included descriptions of risks and benefits, they failed to mention the uncertainty regarding treatment information. With regard to the concreteness of treatment information, “Does it describe how the treatment choices affect the overall quality of life?” (question 13) received the lowest score, indicating that most articles did not refer to the consequences—in particular, quality of life—of no treatment. Thus, the performance with regard to question 13 was similar to that with regard to question 8, reflecting the lack of concreteness, resulting in incomplete information.

As for the content scores according to the Hypertension Guidelines, most (98/130, 75.4%) of the articles lacked key points on hypertension treatment and provided only 2 to 3 key points, leading to the incompleteness of medical treatment content. Medication and lifestyle interventions are frequently referred to in the articles, probably because pharmacological and nonpharmacological interventions (health management) are common ways to treat hypertension [39]. By contrast, the instrument intervention was hardly ever mentioned, probably because of insufficient evidence regarding the efficacy and safety of this method [22].

Nowadays, the supervision of articles published on WeChat mainly concerns legalities, such as network security and legality of the content. Information quality is not yet a concern, and specific measures to ensure information quality are lacking [40,41]. For this, the Health On the Net code of conduct for medical and health websites (HONcode) can provide some references for improvement measures. The HONcode stipulates that all medical advice must come from medical professionals to ensure the authority and accuracy of the information [42]. For the WeChat platform, we need to consider the professional nature of medical health information. The government must strictly review the author’s qualifications as well as the content before publication. In addition, we found that 89.2% (116/130) of the articles had adopted various marketing strategies for promotion of the content, and previous studies have also pointed out this problem. This trend of commercial advertisements disguised as supposedly harmless referral links can become an issue [43]; for example, some publishers exaggerate illness symptoms and product functions to persuade more people to buy drugs and commodities. It is easy to persuade an unsuspecting public that these drugs and products are good for them, but the consequences can be serious in terms of health risks. Therefore, the government must enact strict laws against false advertising with regard to web-based medical information and recommend credible information sources to the public [44].

### Governmental Sources Provided High-Quality Information but Were Lacking in Motivation

In this study, significant differences could be observed among the 3 types of articles and 5 uploading sources in terms of overall quality. With regard to the article types, the differences in quality were mainly due to the quality of the medical treatment content. The adherence to the Hypertension Guidelines was low, the articles lacked key treatment management points, and provided incomplete information. Importantly, with regard to the uploading sources, we found that governmental sources scored significantly higher than individuals. This finding was consistent with prior studies, which suggests that governmental institutions are more likely to publish high-quality information [28]. Presumably because the teams from governmental institutions are highly specialized and knowledgeable, they are more cautious and responsible about what they publish [45]. However, governmental institutions only uploaded 3.1% (4/130) of the articles, which is an indication of poor motivation. As governmental institutions uploaded an insufficient number of articles, we could not arrive at a conclusion regarding their overall performance in the quality score. We know that science is not supposed to be a popularity contest, but governments should exert more effort to disseminate accurate and complete information via social media to ameliorate the negative health consequences of misinformation [46]. By contrast, individuals accounted for 22.3% (29/130) of the articles—presenting high motivation—but the overall quality was not excellent. Noticeably, health care promotion demands high professionalism and strictness, and inaccurate content will mislead the public. Allowing general users to publish content related to highly professional subjects might be inappropriate.

### Articles With References Were of Higher Quality

Citation resources or references can reflect an article’s objectivity to a certain extent [47]; if they are absent, people’s judgment regarding the accuracy of the content as well as their understanding of the health information can be directly affected [36]. In this study, we found that the mean score of articles with references was significantly higher than that of those without, and these scores differed significantly. Of concern, it is not common practice to list references in WeChat articles; only 13.1% (17/130) of the articles listed references. Most (92/130, 70.8%) of the articles provided the author’s name; a few (20/130, 15.4%) provided the author’s name and work credentials. However, an article’s quality and credibility cannot be judged only on the basis of this simple information. Prior studies have suggested that the proportion of content with references was low at 10.2% [45]. Lacking citation resources or references was one of the important factors that led to a severe gap between health information and scientific evidence. The HONcode stipulates traceability, meaning that the content should identify the source of information to which readers could refer [42]. If the WeChat platform identifies the source of the information it publishes, the credibility of this information might be improved. Therefore, the government needs to regulate such articles to ensure that they provide references, perhaps by providing links of citation resources at the end of each article. We found that some WeChat public accounts such as *The Lancet* and *Dingxiang Yisheng* had already done this. Furthermore, we discovered that some articles had provided the number of words in the article and expected reading time, which is a good practice.

### Correlations Among the DISCERN Score, Content Score, Number of Views, and Number of *Likes*

A valuable finding of this study was that there were significant correlations between the DISCERN score and the content score. This indicates that the hypertension treatment articles were more likely to be accurate and complete if they included information about the benefits and risk factors of treatment, the consequences of no treatment, and reference resources, presumably because providing reliable and complete information necessitates using more words and furnishing a detailed explanation. By contrast, there were no significant correlations among the DISCERN score, number of views, and number of *likes*, similar to previous study findings [34]. We found that the more popular articles (high numbers of views and *likes*) were not associated with high DISCERN scores. We believe that the numbers of views and *likes* could objectively reflect the interest of the audience as well as the effect of the operation of the public account, although this has no relation to the quality of the article [48]. Perhaps some of the articles with a larger audience had marketing value or were emotionally charged, making them more appealing to viewers rather than providing reliable knowledge [33].

### Practical Significance

The findings from this study have significant implications for practice. On the one hand, a method that combines the DISCERN tool and Hypertension Guidelines to evaluate information quality is meaningful and comprehensive for social media platforms and future work. On the other hand, if the credibility and concreteness of treatment information were excellent, the medical accuracy, completeness, and overall quality were likely to be better. On the basis of this finding, we advise WeChat users to identify information quality initially according to the following factors: (1) the completeness of the article: if it clearly describes what (the nature of the disease), why (the cause of the disease), and how (treatment) [31]; (2) whether the article provides references or indicates the source of information; and (3) high numbers of views and *likes* do not necessarily mean excellent quality.

Standardizing and managing information from information providers helps to create a harmonious information environment [49]. In this study, we realized the practical significance of the DISCERN tool, and we suggest that authors should consult the DISCERN handbook when writing their article. First, for example, the DISCERN handbook indicates that a good-quality article must have clear aims and achieve the ultimate goal, which is that the title and content should match [27]. In other words, an article should have an appropriate title that expresses the main idea, and the content should be written around this idea. Second, questions 9 to 15 indicate that a good-quality article about disease treatment must focus on the concreteness of the content, such as how each treatment works, the benefits and risks of each treatment, and what would happen if there was no treatment. Third, the DISCERN handbook also reveals that a good-quality article must clarify the source of information; for example, listing the references or sources at the end of the article is a good practice. In brief, if the article is only providing information about a disease, such as hypertension, the author can refer only to DISCERN part 1 (questions 1-8), but if it is also providing information about treatment of the disease, the author should refer to DISCERN part 1 as well as part 2 (questions 1-15). This may be useful with regard to improving the quality of the information.

### Limitations and Future Directions

This study has some limitations. First, we only evaluated the quality of information related to hypertension treatment; thus, the range of the included articles was narrow (only 1 topic). Second, we only focused on 1 platform; there was no comparison among different platforms, leaving an area for further investigation. Future studies should consider including more evaluation dimensions or comparing among different platforms.

### Conclusions

This study is the first to analyze WeChat articles oriented to the general population on hypertension treatment, contributing to a better understanding of the available information on hypertension on WeChat. The evaluation outcomes from the two sources, that is, the DISCERN and the Hypertension Guidelines, show high correlations and suggest valid results. The overall information quality of hypertension treatment–related articles on WeChat was poor. Quality scores differ significantly among the 3 types of articles and 5 uploading sources, revealing the significance of different value propositions. Articles that provided references were of better quality than those that did not. Future work is warranted to regulate information sources and strengthen references. For the treatment of hypertension, crucial information on the consequences of no treatment is urgently needed.

## Appendix

Multimedia Appendix 1 The DISCERN scores for the hypertension treatment information.

## Abbreviations

HONcode: Health On the Net code of conduct for medical and health websites
ICC: intraclass correlation coefficient
