# Supplementary material for: Quality Assessment of Hypertension Treatment–Related Information on WeChat: Cross-sectional Study
Source: J Med Internet Res. 2022 Oct 26;24(10):e38567. doi: 10.2196/38567 (PMC9647448; doi:10.2196/38567)
Supplement: Multimedia Appendix 1 [file jmir_v24i10e38567_app1.docx]

The DISCERN scores for the hypertension treatment information.

| DISCERN Items | Mean Score (SD) | Median | Intraclass Correlation Coefficient (ICC) |
| --- | --- | --- | --- |
| **Part 1: Credibility of information** |  |  |  |
| Q1: Are the aims of the article clear? | 2.87 (0.76) | 3.00 | 0.81 |
| Q2: Does the article achieve its aims? | 2.45 (0.96) | 2.50 | 0.79 |
| Q3: Is the article relevant to patient? | 2.25 (0.83) | 2.00 | 0.79 |
| Q4: Is the article clear what sources of information were used to compile? | 1.70 (1.26) | 1.00 | 0.81 |
| Q5: Is the article clear when the information used or reported was produced? | 1.64 (1.11) | 1.00 | 0.83 |
| Q6: Is the article balanced and unbiased? | 2.54 (0.94) | 3.00 | 0.71 |
| Q7: Does the article provide details of additional sources of support and information? | 1.75 (0.93) | 2.00 | 0.81 |
| Q8: Does the article refer to areas of uncertainty? | 1.38 (0.79) | 1.00 | 0.75 |
| **Part2: Concreteness of treatment information** |  |  |  |
| Q9: Does the article describe how each treatment works? | 2.35 (1.11) | 2.00 | 0.82 |
| Q10: Does the article describe the benefits of each treatment? | 1.97 (0.98) | 2.00 | 0.82 |
| Q11: Does the article describe the risks of each treatment? | 1.53 (0.96) | 1.00 | 0.77 |
| Q12: Does the article describe what would happen if no treatment is used? | 1.19 (0.65) | 1.00 | 0.69 |
| Q13: Does the article describe how the treatment choices affect overall quality of life? | 1.18 (0.46) | 1.00 | 0.76 |
| Q14: Is the article clear that there may be more than one possible treatment choice? | 1.45 (0.75) | 1.00 | 0.77 |
| Q15: Does the article provide support for shared decision-making? | 2.58 (0.93) | 2.50 | 0.76 |
| **Part3: Overall subject quality** |  |  |  |
| Q16: Based on the answers to all of the above questions, rate the overall quality of the article as a source of information about treatment choices. | 2.40 (0.86) | 2.00 | 0.76 |
| **Sum Score** | 31.22 (8.46) | 30.00 | 0.97 |
